# Maternal smoking during pregnancy and offspring intellectual disability: sibling analysis in an intergenerational Danish cohort

**DOI:** 10.1017/S0033291720003621

**Published:** 2020-10-14

**Authors:** Paul Madley-Dowd, Amy E. Kalkbrenner, Hein Heuvelman, Jon Heron, Stanley Zammit, Dheeraj Rai, Diana Schendel

**Affiliations:** 1 Centre for Academic Mental Health, Population Health Sciences, Bristol Medical School, University of Bristol, Bristol, UK; 2 NIHR Biomedical Research Centre, University of Bristol, Bristol, UK; 3 Joseph J. Zilber School of Public Health, University of Wisconsin-Milwaukee, Milwaukee, Wisconsin, USA; 4 MRC Centre for Neuropsychiatric Genetics and Genomics, Cardiff University, Cardiff, UK; 5 Avon and Wiltshire Partnership NHS Mental Health Trust, Bristol, UK; 6 The Lundbeck Foundation Initiative for Integrative Psychiatric Research (iPSYCH), Aarhus, Denmark; 7 National Centre for Register-based Research, Department of Economics and Business, Aarhus University, Aarhus, Denmark; 8 Department of Public Health, Aarhus University, Aarhus, Denmark

**Keywords:** Confounding, intellectual disability, maternal smoking, neurodevelopment, sibling design, tobacco

## Abstract

**Background.:**

Maternal smoking has known adverse effects on fetal development. However, research on the association between maternal smoking during pregnancy and offspring intellectual disability (ID) is limited, and whether any associations are due to a causal effect or residual confounding is unknown.

**Method.:**

Cohort study of all Danish births between 1995 and 2012 (1 066 989 persons from 658 335 families after exclusions), with prospectively recorded data for cohort members, parents and siblings. We assessed the association between maternal smoking during pregnancy (18.6% exposed, collected during prenatal visits) and offspring ID (8051 cases, measured using ICD-10 diagnosis codes F70–F79) using logistic generalised estimating equation regression models. Models were adjusted for confounders including measures of socio-economic status and parental psychiatric diagnoses and were adjusted for family averaged exposure between full siblings. Adjustment for a family averaged exposure allows calculation of the within-family effect of smoking on child outcomes which is robust against confounders that are shared between siblings.

**Results.:**

We found increased odds of ID among those exposed to maternal smoking in pregnancy after confounder adjustment (OR 1.35, 95% CI 1.28–1.42) which attenuated to a null effect following adjustment for family averaged exposure (OR 0.91, 95% CI 0.78–1.06).

**Conclusions.:**

Our findings are inconsistent with a causal effect of maternal smoking during pregnancy on offspring ID risk. By estimating a within-family effect, our results suggest that prior associations were the result of unmeasured genetic or environmental characteristics of families in which the mother smokes during pregnancy.

## Background

Smoking in pregnancy is reported in at least 10% of pregnancies in many European countries as of 2010 (European Perinatal Health Report, 2013). It has well-established associations with poor offspring health outcomes such as low birthweight (Davey Smith, 2008; Tyrrell et al., 2012). Establishing whether maternal smoking in pregnancy is causally related to other offspring health outcomes may provide insight as to which disease burdens may be reduced through smoking cessation initiatives and may provide mechanistic insights into the causes of these conditions. Although the best way to establish causality is to use an experimental design, this is not ethical with maternal smoking outside of a smoking cessation intervention. Observational analyses using readily available data that attempt to account for confounding and biasing factors are the most appropriate alternative.

Nicotine, the psychoactive component in tobacco smoke, has been shown to cross the placenta and expose the fetus to higher concentrations than the mother (Jauniaux, Gulbis, Acharya, Thiry, & Rodeck, 1999; Luck, Nau, Hansen, & Steldinger, 1985). Once nicotine has reached the fetus, it acts on nicotinic acetylcholine receptors. Animal models suggest that this influences developmental processes in the brain including neurogenesis, migration, differentiation, and synaptogenesis (Dwyer, Broide, & Leslie, 2008). The many other toxic constituents of smoking may also reach the fetal brain and influence development. Associations in humans between maternal smoking in pregnancy and changes to offspring brain morphology have been found using measures such as reduced head circumference (Ekblad, Korkeila, & Lehtonen, 2015) and fetal brain volumes (Anblagan et al., 2013; Roza et al., 2007). More recent studies using magnetic resonance imaging have found differences in the size of the corpus callosum of 9–11-year-olds between exposed and unexposed individuals, suggesting that maternal smoking during pregnancy may have long-term effects on brain structure (Biffen et al., 2017).

It is plausible that smoking-induced brain changes could influence neurodevelopmental outcomes. Associations between exposure to maternal smoking during pregnancy have consistently been found for poor academic achievement and behavioural problems in children (Clifford, Lang, & Chen, 2012; Polanska, Jurewicz, & Hanke, 2015), in particular Attention Deficit Hyperactivity Disorder (ADHD) (Huang et al., 2018). Less consistent findings have been found for offspring intelligence, memory, attention and executive function. In recent years evidence has been collated to suggest a lack of association between smoking in pregnancy and offspring Autism Spectrum Disorders (ASD) (Jung, Lee, McKee, & Picciotto, 2017; Kalkbrenner et al., 2019; Rosen, Lee, Lee, Yang, & Burstyn, 2015; Tang, Wang, Gong, & Wang, 2015; Wang, Geng, Liu, & Zhang, 2017).

Whether any observed associations reflect a causal relationship remains unclear. There are several strong risk factors associated with neurodevelopmental outcomes that are also correlated with maternal smoking in pregnancy. Such variables are potential confounders that may bias association estimates. These include socioeconomic status (Hair, Hanson, Wolfe, & Poliak, 2015; Lu, Tong, & Oldenburg, 2001), parity (Belmont & Marolla, 1973; Lu et al., 2001), year of birth (Cnattingius, 2004; Polanczyk, Willcutt, Salum, Kieling, & Rohde, 2014), parental education (Cnattingius, 2004; Eriksen et al., 2013; Lu et al., 2001), age at birth (Lu et al., 2001; Merikangas et al., 2017), psychiatric history (Goodwin, Keyes, & Simuro, 2007; Gutierrez-Galve, Stein, Hanington, Heron, & Ramchandani, 2015), and immigration status (Abdullahi et al., 2018; Melchior et al., 2015).

Most research in offspring cognitive effects of maternal smoking to date has focused on variation in IQ within the normal range. Less is known about the potential impact of smoking during pregnancy on the risk of more severe and debilitating cognitive impairments, such as those present in intellectual disability (ID). ID is defined as having an IQ of less than 70 alongside functional impairment (World Health Organization, 2018). Individuals with ID suffer from poor long-term outcomes and inequalities compared to the general population such as worse access to and effectiveness of health care (Lennox & Kerr, 1997; Michael, 2008; Whitfield, Langan, & Russell, 1996; Wilson & Haire, 1990), and increased mortality (Hosking et al., 2016) and socioeconomic disadvantage (Emerson, Einfeld, & Stancliffe, 2010; Hassiotis et al., 2008).

A systematic review has suggested that smoking during pregnancy is associated with a small increase in the risk of offspring ID (Huang, Zhu, Qu, & Mu, 2016). Evidence not included in the review, but that made better attempts to account for bias than the included studies, suggested that the association may be the result of confounding. Braun, Daniels, Kalkbrenner, Zimmerman, and Nicholas (2009) used data linkage in a North American surveillance cohort to control for confounding by aggregated area level socioeconomics while Lundberg et al. (2010) used a sibling design in a cohort of Swedish male conscripts to account for unmeasured shared familial confounding. Neither study found evidence consistent with a causal effect. These findings may point to the role of unmeasured confounding in the association reported in the systematic review.

There is some evidence to suggest that the elevated risk associated with maternal smoking in pregnancy is limited to specific subgroups. One analysis has suggested that the association was specific to males who were exposed to more than 20 cigarettes smoked per day (Braun et al., 2009). A separate study found an increased risk of offspring ID for those with longer but not shorter gestational lengths (Hirvonen et al., 2017), possibly suggesting a sensitive period of exposure in late gestation aligning with specific events of foetal brain development (Andescavage et al., 2017; Bouyssi-Kobar et al., 2016). Neither analysis, however, adequately accounted for confounding.

Taken together, the current literature raises an important question about whether observed associations may be accounted for by confounding, though the evidence is inconclusive. Furthermore, important questions are outstanding, such as whether the association between maternal smoking and offspring ID differs by offspring gender or with the presence of other comorbid disorders, and whether timing and dosage of exposure are associated with changes in the strength of association. The goals of this study were to investigate the association between maternal smoking during pregnancy and risk of ID in offspring and assess causality, using data from a large Danish population-based cohort with data available on parents and siblings. Secondary aims were to investigate the association among subgroups (separated by severity of ID, comorbid ASD and ADHD, gender) and the associations for different timings and dosages of exposure.

## Methods

### Ethics approval

This study was approved by the Danish Scientific Ethics Committee, the Danish Health Data Authority, the Danish Data Protection Agency and the Danish Neonatal Screening Biobank Steering Committee. Consent from individuals for this register-based study using anonymised data was not required.

### Cohort for analysis

**Fig. 1.**
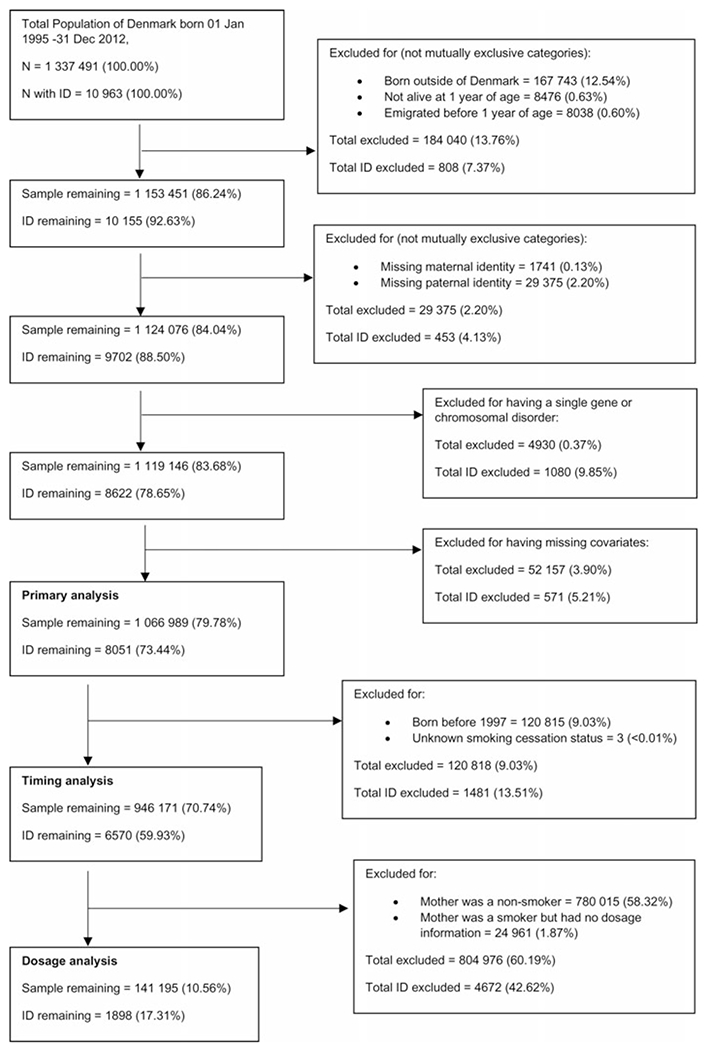
Flowchart of cohort derivation.

The study cohort consisted of all individuals born in Denmark between 1 January 1995 and 31 December 2012 (*n* = 1 337 491). After excluding children not born in Denmark, those who died or emigrated before the age of 1, those who had a missing link to a maternal or paternal identifier and those who had a known genetic or metabolic cause of intellectual disability (see online supplementary Table S1), the remaining sample included 1 119 146 individuals (study flow chart in Fig. 1). The cohort and analytic variables were defined using several registry datasets linked by a unique identification number (Schmidt, Pedersen, & Sorensen, 2014): the Danish Medical Birth Registry (MBR) (Bliddal, Broe, Pottegard, Olsen, & Langhoff-Roos, 2018), the Danish Psychiatric Registry (DPR) (Mors, Perto, & Mortensen, 2011), the Danish National Patient Registry (NPR) (Schmidt et al., 2015) and Statistics Denmark registries of education (Jensen & Rasmussen, 2011) and income (Baadsgaard & Quitzau, 2011).

Most clinical contacts related to intellectual disability occurred in an outpatient setting. We, therefore, defined the start year of the cohort as 1995 when the DPR and NPR started recording outpatient contact in addition to inpatient admissions. We selected 2012 as the end year for inclusion in the cohort to allow a minimum of 4 years follow up until the latest date for available data, 10 April 2017. The youngest and oldest members of the cohort were followed up until approximately 4.3 and 22.3 years of age, respectively.

### Exposure definition: maternal smoking during pregnancy

Information about maternal smoking during pregnancy was obtained from the MBR, abstracted from midwife interviews at the first antenatal contact. A dichotomous smoking variable (yes/no) is available for births between 1995 and 1996. From 1997 additional information on duration (i.e. whether they stopped smoking and whether smoking cessation was before or after the first trimester) and number of cigarettes smoked per day (up to 5 cigarettes, 6–10 cigarettes, 11–20 cigarettes, >20 cigarettes) was added. Reporting of the additional duration and dosage data did not occur until late 1997 and took a few years to reach >95% completeness (Bliddal et al., 2018). We created a categorical timing variable among those born from 1997 onwards with available data (*n* = 946 171) that indicated whether mothers did not smoke during pregnancy, smoked but gave up before the end of the first trimester or smoked beyond the end of the first trimester. Finally, we created a continuous dosage variable, where data were available (*n* = 141 195), that indicated the number of cigarettes smoked per day using the lower bound of the dosage groups (i.e. 1, 6, 11 or 21 cigarettes smoked per day).

The validity of the MBR smoking measure is supported by correlations between the MBR maternal smoking data and biomarkers of smoking-related methylation in new-born offspring (Hannon et al., 2018). We assessed the reliability of our dichotomous smoking measure by comparing smoking status during pregnancy in the MBR against the NPR (see the online appendices; section A.1).

### Outcome definitions: intellectual disability

ID was defined as having an ICD-10 (World Health Organization, 2018) code of F70–F79, recorded as a primary or secondary diagnosis in either the DPR or the NPR. After exclusions (see Fig. 1), 8051 cases (0.75% of included persons during the included follow-up period) were identified.

### Comorbid neurodevelopmental disorder definitions:

Matching the definition used by the Lundbeck Foundation Initiative for Integrative Psychiatric Research (iPSYCH) consortium (Pedersen et al., 2018), individuals with the ICD-10 diagnosis codes F84.0, F84.1, F84.5, F84.8 and F84.9 were identified as having ASD. Individuals with the diagnosis codes F90.0 were identified as having an Attention Deficit Hyperactivity Disorder (ADHD). Where iPSYCH used diagnoses from only the DPR, we also used diagnoses from the NPR.

### Covariate and confounder definitions

The covariates and confounders adjusted for in statistical models were child sex, parity, mother and father’s age, education and income in the year of the child’s birth, the psychiatric history of mother and father prior to the child’s birth and mother and father’s country of origin.

Highest educational attainment of either parent was obtained from Danish education registers and derived into a categorical variable separated into primary education (6–16-years-old), general/vocational education (post 16 education) and higher education (university level of any duration). Parental income was obtained from the Statistics Denmark registry of income. We derived a measure of household income, adjusted for family size, in deciles for each year (to account for inflation). Parental country of origin was classified as belonging to the following locations: Denmark (including Greenland), Africa, Americas, Europe, Middle East, Oceania (Asia and Australia) and Scandinavia.

For parental psychiatric history, we derived indicator variables for diagnoses of affective disorders, anxiety disorders, psychotic disorders and substance use disorders (excluding nicotine-related disorders; ICD-10 code F17) in either the DPR or the NPR for each parent at any time before the child’s birth. As the diagnostic system used in Denmark changed from ICD-8 to ICD-10 in 1994 we used the conversion table presented by Pedersen et al. (2014) to convert between the two classification systems. The diagnosis codes used to derive the indicator variables are presented in online supplementary Table S2.

### Assessment of missing data

There were little missing data (overall 3.9%, 52 157 individuals) due to missing exposure, confounder or covariate variables. We present our missing data assessment in the online appendices (methods: section A.2; results: section B.1).

### Statistical analysis

All analyses were performed using R version 3.4.3 (R Core Team, 2017). Following descriptive analyses, our primary analyses involved logistic regression of ID on maternal smoking in pregnancy. The family structure present within the cohort means that the data violates the assumption of independence between observations which can lead to underestimation of standard errors. We, therefore, used generalised estimating equations (GEE) (Carey, Lumley, Ripley, & Moler, 2015), with an exchangeable correlation structure for mother and father combinations. This means our analyses accounted for correlations between full siblings, but half-siblings, cousins, and other relations were treated as independent. All models (including those referred to as unadjusted) were adjusted for child’s grouped year of birth (1995–1997, 1998–2000, 2001–2003, 2004–2006, 2007–2009, 2010–2012) to account for cohort effects and the differing length of follow up across birth years.

### Primary analysis

We ran four models. Model 1 was not adjusted for any further covariates. Model 2 was adjusted for covariates and confounders. Model 3 adjusted for family-level smoking by including a term equal to the proportion of pregnancies in the family in which the mother was recorded as having smoked, thus making use of model formulation 2 suggested by Begg and Parides (2003), but without other covariates. Model 4 adjusted for all covariates, confounders and the family-level smoking variable.

Adjustment for family-level smoking, as in Model 3 and 4, allows the calculation of within-family (coefficient of the individual level exposure) and between-family (coefficient of the family-level exposure) effects of smoking on ID. The within-family effect is robust against confounders that are shared between the siblings. Failing to find a within-family effect after adjustment for the family-averaged exposure variable is consistent with familial confounding and there being no causal effect of the exposure on the outcome (Begg & Parides, 2003; Carlin, Gurrin, Sterne, Morley, & Dwyer, 2005).

### Positive control analysis

To test the validity of this approach we performed a positive control analysis in which we repeated the analyses using birthweight instead of ID, an outcome that is well established as having a causal relationship with maternal smoking in pregnancy. We repeated the four models using low birthweight as the outcome, defined as a birthweight of less than 2500 g (4.73% of included persons). Birthweight (mean value = 3948 g, s.d. = 590 g) was obtained from the MBR for 1 062 474 individuals (99.6% of the primary analysis sample).

### Secondary and sensitivity analyses

In secondary analyses, we assessed the association between maternal smoking and offspring ID for different severities of ID and comorbidities of ID with ADHD and ASD. We also assessed differences in effect size based on sex, smoking timing, and dosage. In sensitivity analyses, we assessed whether results were robust to (i) measurement error in the outcome; (ii) differing lengths of follow up between cohort years; and (iii) potential biases arising from smoking patterns in the cohort. Details of how these analyses were performed can be found in the online appendices (section A.3).

## Results

### Description of the cohort

Characteristics of the study cohort are displayed in Table 1. Maternal smoking was reported in 18.6% of pregnancies and was associated with lower maternal and paternal age at pregnancy, lower parental education, being in a lower decile of income, and increased parity. All psychiatric disorders were more common in smokers and their partners compared to families in which the mother did not smoke during pregnancy. The prevalence of maternal smoking during pregnancy decreased over time. Further patterns of smoking and ID in the cohort are described in the online appendices (sections B.2 and B.3 respectively).

### Primary analyses of the association between maternal smoking and offspring ID

Maternal smoking during pregnancy was associated with increased odds of ID in unadjusted analysis (Table 2; OR 1.91, 95% CI 1.82–2.00). This was attenuated following adjustment for covariates and confounders (OR 1.35, 95% CI 1.28–1.42). The within-family effect, obtained from the model adjusted for the family-level smoking variable, was found to be null before and after adjustment for confounders; before (OR 0.91, 95% CI 0.78–1.06), after (OR 0.93, 95% CI 0.79–1.09). The between-family effect showed increased odds of ID before and after confounder adjustment.

### Positive control analyses of the association between maternal smoking and offspring low birthweight

In our positive control analyses (see Table 3) we found that maternal smoking in pregnancy was associated with increased odds of low offspring birthweight that was slightly attenuated after adjustment for confounders. Both the within-family effect and between-family effect showed notable attenuation of the association between maternal smoking and low birthweight, although all estimates remained consistent with increased odds of low birthweight before and after confounder adjustment.

### Secondary analyses

We present only the key findings of our secondary analyses. Full details are provided in the online appendices (section B.4.1). Results did not substantially differ when the outcome was ID only compared to when the outcome was ID with ASD or ID with ADHD (online supplementary Table S3 for ASD and Table S4 for ADHD). We found no evidence for an interaction between smoking in pregnancy and offspring sex (online supplementary Table S5). After adjustment for confounders, stopping smoking in the first trimester did not differ from not smoking during pregnancy in terms of odds of ID, whereas continuing smoking after the first trimester showed increased odds of ID (online supplementary Table S6). Dosage analyses showed increased odds of ID for every cigarette smoked per day in conventional adjusted analyses, however, the within-family effect was null (online supplementary Table S7).

### Sensitivity analyses

**Fig. 2.**
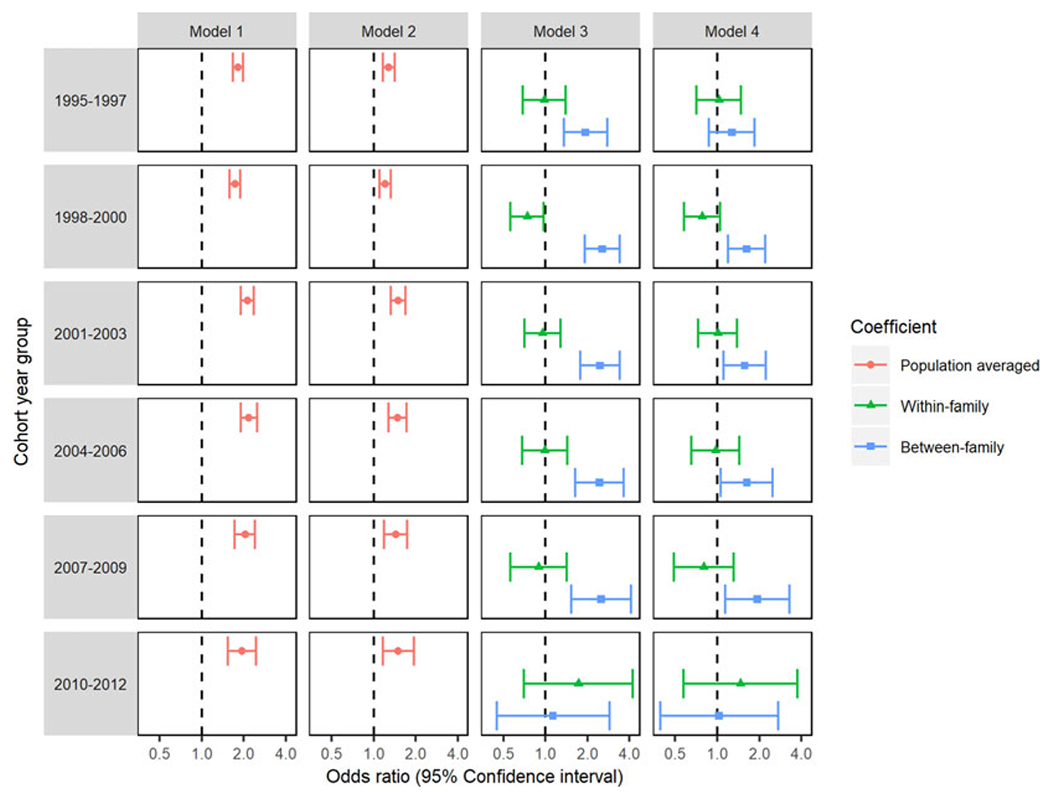
Logistic GEE analyses of the association between maternal smoking during pregnancy and offspring ID repeated in each cohort year group.

Our sensitivity analyses (described in more detail in the online appendices; section B.4.2) showed that our results were not substantially influenced by using a stricter definition of the outcome variable (online supplementary Table S8) or by differing lengths of follow up between cohorts (see Fig. 2 for models stratified by cohort year group and online supplementary Table S9 for results of time-to-event models). The results of analyses in a sample restricted to single-child families showed lower adjusted ORs for smoking in pregnancy compared to the primary analyses (see online supplementary Table S10), whereas, in a sample restricted to multiple child families, results were comparable to those of the primary analyses.

## Discussion

Using a large population-based cohort we have provided evidence that the association between maternal smoking during pregnancy and offspring ID is not consistent with a causal relationship. Instead, the association appears to be driven by residual confounding. Our primary analyses showed that when population-averaged associations were decomposed into within-family and between-family effects there was no influence of individual exposure to smoking in pregnancy on the risk of ID. Associations were instead driven by family-level differences in maternal smoking behaviour.

The consistency of results between primary and sensitivity analyses provide evidence that our conclusions were robust to (i) measurement error in the outcome, (ii) differing lengths of follow up between cohort year groups and (iii) potential biases arising from patterns of smoking in the cohort. Our results were also not influenced by comorbidities of ID with ASD or with ADHD. We validated our analysis approach by performing a positive control analysis in which low birthweight was used as an outcome. Here a causal relationship was expected (Davey Smith, 2008; Tyrrell et al., 2012). We found a small within-family effect suggesting that once family-level differences in exposure were accounted for there was a small increase in the risk of low birthweight for those exposed to smoking in pregnancy.

It is likely that associations with maternal smoking in pregnancy reported in prior studies were due to residual confounding. Braun et al. (2009) found strong attenuation of their confounder adjusted association following further adjustment for area-level socioeconomic information obtained from data linkage. Comparison with our study suggests that linked data are not always sufficient to account for confounding structures. Further accounting for family structure as in our analyses that estimated a within-family effect, and analyses performed by Lundberg et al. (2010), demonstrate that associations between maternal smoking in pregnancy and offspring ID are unlikely to be causal. This is consistent with the results of other studies using family-based designs on cognitive and neurodevelopmental outcomes such as academic achievement, general cognitive ability, and conduct problems (D’Onofrio et al., 2008, 2010; Kuja-Halkola, D’Onofrio, Larsson, & Lichtenstein, 2014). It should be noted that these methods have demonstrated potential causal effects of maternal smoking during pregnancy on adverse pregnancy outcomes including birthweight, preterm birth, and being born small for gestational age.

### Study strengths and limitations

Our study strengths include our large population-based sample size which reduced the risk of selection bias and improved generalisability. Extensive data linkage allowed for adjustment for many confounding variables and for the derivation of family-level exposure variables which allowed for the understanding of residual confounding.

Our study has several limitations. We used registry data, and some misclassification in the recording of exposures and outcomes cannot be ruled out. This would be a limitation in any large-scale record linkage study. We had information on a range of potential confounding factors although we were unable to study the role of some potentially relevant factors such as gestational diet quality, alcohol or substance misuse during pregnancy or the role of passive smoke exposure to the mother during pregnancy, or to the child following birth. Although there were little missing data (3.9%), since those excluded were also more likely to have ID, our complete case analysis may be biased towards the null compared to a fully observed dataset. Finally, we note that the reported between-family effect is likely to be imprecise due to the small cluster size of families (Begg & Parides, 2003).

### Further research

The between-family effect showed increased odds of ID for families in which the mother tended to smoke in more pregnancies, holding fixed individual-level exposure to smoking. Exploring the factors that vary systematically between families that influence both smoking during pregnancy and offspring ID would inform further research as to what additional variables need to be adjusted for in analyses and may also guide targets for public health interventions. Quasi-experimental methods that exploit family structure to vary the degree to which individuals are genetically similar to one another have been developed (D’Onofrio, Lahey, Turkheimer, & Lichtenstein, 2013). These studies compare estimates of within-family effects for families defined by increasingly dissimilar genetic relatedness (e.g. full siblings, half-siblings, offspring of full sisters and offspring of half-sisters) to provide evidence as to the extent that residual confounding is genetic and environmental in nature.

Instrumental variable approaches such as Mendelian Randomisation (Davey Smith & Ebrahim, 2003; Davey Smith & Hemani, 2014; Lawlor et al., 2017) may also be informative in the exploration of the residual confounding structure as they would specifically assess the influence of the mother’s predisposition to smoking behaviours. This is likely to be comparable to our family-level smoking variable. The family-level exposure variable may, however, reflect genetic confounding in that a genetic propensity for maternal smoking may be associated with offspring ID via pleiotropic mechanisms rather than via maternal smoking in pregnancy. For example, polygenic risk scores (PRS) for ADHD, which are likely to correlate with genetic risk of ID due to the high prevalence of comorbidity, have been found to predict smoking behaviour (Demontis et al., 2019; Leppert et al., 2019). In this case, standard Mendelian Randomisation would not be appropriate due to violation of the exclusion restriction criteria (the assumption that an instrument is associated with the outcome only via the exposure) (Davies, Holmes, & Davey Smith, 2018) and extensions such as multivariable Mendelian Randomisation would be required (Burgess & Thompson, 2015).

## Conclusions

Based on the consistent findings of no association between maternal smoking and ID across the primary, secondary, and sensitivity analyses in conventional and family-based analytic approaches, this study provides evidence against a causal effect of maternal smoking during pregnancy on offspring intellectual disability. The persistent between-family effect in the absence of a within-family effect in adjusted analyses provides evidence in support of the role of residual confounding. A lack of causal effect of maternal smoking in pregnancy on offspring ID should not be interpreted as meaning that smoking in pregnancy is safe. It has a range of other demonstrable negative health consequences and these results should not distract from the sustained efforts required to reduce its prevalence.

## Supplementary Material

Supplement 2

Supplement 1

## Figures and Tables

**Table 1.** Characteristics of the sample by maternal smoking during pregnancy (exposure) status

Characteristic	Smokers	Non-smokers	*p* value^a^
Total, *N* (%)	198 377 (18.6)	868 612 (81.4)	
Maternal age, mean (s.d.)	28.7 (5.26)	30.1 (4.64)	<0.001
Paternal age, mean (s.d.)	31.4 (6.16)	32.7 (5.58)	<0.001
Highest parental education, *N* (%)			<0.001
Primary	46 456 (23.4)	60 817 (7.0)	
General/vocational	110 361 (55.6)	351 798 (40.5)	
Higher	41 560 (21.0)	455 997 (52.5)	
Income decile, median (IQR)	4 (1–6)	5 (2–7)	<0.001
Maternal country of origin, *N* (%)			<0.001
Denmark	181 453 (91.5)	746 737 (86.0)	
Africa	844 (0.4)	16 286 (1.9)	
Americas	700 (0.4)	5313 (0.6)	
Europe	8474 (4.3)	37 821 (4.4)	
Middle East	2000 (1.0)	23 403 (2.7)	
Oceana	2045 (1.0)	27 073 (3.1)	
Scandinavia	2861 (1.4)	11 979 (1.4)	
Paternal country of origin, *N* (%)			<0.001
Denmark	180 019 (90.7)	751 091 (86.5)	
Africa	1339 (0.7)	17 641 (2.0)	
Americas	728 (0.4)	4898 (0.6)	
Europe	10 165 (5.1)	39 870 (4.6)	
Middle East	2989 (1.5)	27 359 (3.1)	
Oceana	1162 (0.6)	18 884 (2.2)	
Scandinavia	1975 (1.0)	8869 (1.0)	
Maternal Psychiatric history, *N* (%)			
Affective disorder	5398 (2.7)	12 945 (1.5)	<0.001
Anxiety disorder	12 527 (6.3)	27 332 (3.1)	<0.001
Psychotic disorder	1953 (1.0)	3119 (0.4)	<0.001
Substance use disorder	8433 (4.3)	9587 (1.1)	<0.001
Paternal Psychiatric history, *N* (%)			
Affective disorder	1868 (0.9)	4840 (0.6)	<0.001
Anxiety disorder	5245 (2.6)	12 158 (1.4)	<0.001
Psychotic disorder	1440 (0.7)	3537 (0.4)	<0.001
Substance use disorder	9554 (4.8)	15 985 (1.8)	<0.001
Child sex, *N* (%)			0.22
Female	96 406 (48.6)	423 450 (48.8)	
Male	101 971 (51.4)	445 162 (51.2)	
Parity, *N* (%)			<0.001
0	85 111 (42.9)	376 648 (43.4)	
1	69 489 (35.0)	331 010 (38.1)	
2	31 007 (15.6)	121 489 (14.0)	
3 +	12 770 (6.4)	39 465 (4.5)	
Cohort year, *N* (%)			<0.001
1995–1997	47 205 (23.8)	133 189 (15.3)	
1998–2000	42 271 (21.3)	139 861 (16.1)	
2001–2003	34 592 (17.4)	143 239 (16.5)	
2004–2006	29 781 (15.0)	150 893 (17.4)	
2007–2009	24 601 (12.4)	153 931 (17.7)	
2010–2012	19 927 (10.0)	147 499 (17.0)	

a *t* tests were performed for normally distributed continuous variables, Wilcoxon rank sum tests were performed for non-normally distributed continuous variables, and χ^2^ tests were performed for binary/categorical variables.

**Table 2.** Primary analysis of the association between maternal smoking and offspring intellectual disability

Model	Coefficient	O.R.	95% CI
Unadjusted	Population averaged	1.91	1.82–2.00
Adjusted for confounders^a^	Population averaged	1.35	1.28–1.42
Adjusted for family smoking variable	Within-family	0.91	0.78–1.06
	Between-family	2.25	1.92–2.63
Adjusted for confounders^a^ and family smoking variable	Within-family	0.93	0.79–1.09
	Between-family	1.51	1.28–1.79

a Adjusted for child sex, parity and year of birth, mother and father’s age, education and income in the year of the child’s birth, the psychiatric history of mother and father prior to the child’s birth and mother and father’s country of origin.

**Table 3.** Positive control analysis of the association between maternal smoking and offspring low birthweight

Model	Coefficient	O.R.	95% CI
Unadjusted	Population averaged	1.88	1.85–1.92
Adjusted for confounders^a^	Population averaged	1.74	1.70–1.77
Adjusted for family smoking variable	Within-family	1.21	1.14–1.27
	Between-family	1.64	1.54–1.74
Adjusted for confounders^a^ and family smoking variable	Within-family	1.06	1.00–1.13
	Between-family	1.73	1.61–1.85

a Adjusted for child sex, parity and year of birth, mother and father’s age, education and income in the year of the child’s birth, the psychiatric history of mother and father prior to the child’s birth and mother and father’s country of origin.
