# Supplementary material for: Maternal smoking during pregnancy and offspring intellectual disability: sibling analysis in an intergenerational Danish cohort
Source: Psychol Med. 2020 Oct 14;52(10):1847–56. doi: 10.1017/S0033291720003621 (PMC8044256; doi:10.1017/S0033291720003621)
Supplement: Supplementary file 1 [file S0033291720003621sup.zip › S0033291720003621sup001.docx]

Supplementary tables

Maternal smoking during pregnancy and offspring intellectual disability: sibling analysis in an intergenerational Danish cohort

Contents

[Table S1: ICD-10 diagnoses used for exclusion 2](#_Toc48647252)

[Table S2: ICD-8 and ICD-10 codes used to define parental psychiatric history 3](#_Toc48647253)

[Table S3: Multinomial logistic GEE analyses of the association between maternal smoking in pregnancy and each category of ID and ASD comorbidity 4](#_Toc48647254)

[Table S4: Multinomial logistic GEE analyses of the association between maternal smoking in pregnancy and each category of ID and ADHD comorbidity 5](#_Toc48647255)

[Table S5: Logistic GEE analyses of the association between maternal smoking during pregnancy and offspring ID, including an interaction between maternal smoking and offspring sex 6](#_Toc48647256)

[Table S6: Logistic GEE analyses of the association between maternal smoking cessation in pregnancy and offspring ID 7](#_Toc48647257)

[Table S7: Logistic GEE analyses of the association between dosage of maternal smoking during pregnancy and offspring ID 8](#_Toc48647258)

[Table S8: Logistic GEE analyses of the association between maternal smoking during pregnancy and offspring ID using a stricter outcome definition 9](#_Toc48647259)

[Table S9: Cox proportional hazards analyses of the association between maternal smoking during pregnancy and time to offspring first diagnosis of ID 10](#_Toc48647260)

[Table S10: Analyses of the association between maternal smoking during pregnancy and offspring ID repeated in restricted cohorts 11](#_Toc48647261)

# Table S1: ICD-10 diagnoses used for exclusion

| ICD-10 Code | Description |
| --- | --- |
| Q89.8 | Williams syndrome |
| Q87.1 | Prader-Willi syndrome |
| Q87.2 | Rubinstein-Taybi syndrome |
| Q44.7 | Alagille syndrome |
| D82.1 | DiGeorge syndrome |
| Q85.0 | Neurofibromatosis (non-malignant) |
| Q85.1 | Tuberous sclerosis |
| Q90-Q99 | Chromosomal abnormalities, not elsewhere specified |
| E70-E72 | Metabolic disorders |

# Table S2: ICD-8 and ICD-10 codes used to define parental psychiatric history

| Disorder | ICD-8 codes | ICD-10 codes |
| --- | --- | --- |
| Anxiety disorders | 300.x9 (excluding 300.49), 305.x9, 305.68, 307.99 | F40-F48 |
| Depressive disorders | 296.09 ,296.29, 298.09, 300.49 | F32-F39 |
| Affective and non-affective psychoses | 295.x9, 296.39, 296.89, 297.x9, 298.29-298.99, 299.04, 299.05, 299.09, 301.83, 296.19, 298.19 | F20-F29, F30-F31 |
| Substance use disorders | 291.x9, 294.39, 303.x9, 303.20, 303.28, 303.90, 304.x9 | F10-F16, F18-F19 |

# Table S3: Multinomial logistic GEE analyses of the association between maternal smoking in pregnancy and each category of ID and ASD comorbidity

|  |  | ID only | | ASD only | | ID + ASD | |
| --- | --- | --- | --- | --- | --- | --- | --- |
| Model | Coefficient | O.R. | 95% CI | O.R. | 95% CI | O.R. | 95% CI |
| Unadjusted | - Population averaged | 2.21 | 2.09 - 2.34 | 1.37 | 1.32 - 1.42 | 1.40 | 1.29 - 1.53 |
| Adjusted for confounders ^a^ | - Population averaged | 1.42 | 1.33 - 1.51 | 1.24 | 1.20 - 1.29 | 1.21 | 1.10 - 1.32 |
| Adjusted for family smoking variable | - Within-family | 0.88 | 0.74 - 1.05 | 1.16 | 1.05 - 1.29 | 0.98 | 0.75 - 1.28 |
|  | - Between-family | 2.76 | 2.29 - 3.32 | 1.21 | 1.09 - 1.35 | 1.50 | 1.13 - 1.99 |
| Adjusted for confounders ^a^ and family smoking variable | - Within-family | 0.91 | 0.76 - 1.10 | 1.07 | 0.96 - 1.19 | 0.96 | 0.73 - 1.28 |
|  | - Between-family | 1.63 | 1.33 - 1.98 | 1.19 | 1.06 - 1.34 | 1.29 | 0.95 - 1.74 |

All odds ratios are relative to the group with no ID or ASD ^a^ Adjusted for child parity and year of birth, mother and father’s age, education and income in the year of the child’s birth, the psychiatric history of mother and father prior to the child’s birth and mother and father’s country of origin.

# Table S4: Multinomial logistic GEE analyses of the association between maternal smoking in pregnancy and each category of ID and ADHD comorbidity

|  |  | ID only | | ADHD only | | ID + ADHD | |
| --- | --- | --- | --- | --- | --- | --- | --- |
| Model | Coefficient | O.R. | 95% CI | O.R. | 95% CI | O.R. | 95% CI |
| Unadjusted | - Population averaged | 1.76 | 1.66 - 1.86 | 2.27 | 2.20 - 2.34 | 2.43 | 2.22 - 2.66 |
| Adjusted for confounders ^a^ | - Population averaged | 1.30 | 1.22 - 1.38 | 1.63 | 1.58 - 1.69 | 1.54 | 1.40 - 1.70 |
| Adjusted for family smoking variable | - Within-family | 0.92 | 0.77 - 1.09 | 0.99 | 0.90 - 1.09 | 0.88 | 0.67 - 1.16 |
|  | - Between-family | 2.07 | 1.72 - 2.48 | 2.56 | 2.31 - 2.83 | 3.12 | 2.33 - 4.17 |
| Adjusted for confounders ^a^ and family smoking variable | - Within-family | 0.94 | 0.78 - 1.13 | 0.95 | 0.86 - 1.05 | 0.90 | 0.67 - 1.21 |
|  | - Between-family | 1.44 | 1.19 - 1.75 | 1.86 | 1.67 - 2.07 | 1.84 | 1.35 - 2.50 |

All odds ratios are relative to the group with no ID or ADHD ^a^ Adjusted for child parity and year of birth, mother and father’s age, education and income in the year of the child’s birth, the psychiatric history of mother and father prior to the child’s birth and mother and father’s country of origin.

# Table S5: Logistic GEE analyses of the association between maternal smoking during pregnancy and offspring ID, including an interaction between maternal smoking and offspring sex

| Model | Coefficient | O.R. | 95% CI |
| --- | --- | --- | --- |
| Unadjusted | - Smoking in pregnancy | 1.93 | 1.78, 2.10 |
|  | - Male sex | 1.93 | 1.82, 2.04 |
|  | - Interaction term | 0.98 | 0.89, 1.08 |
| Adjusted for confounders ^a^ | - Smoking in pregnancy | 1.37 | 1.26, 1.49 |
|  | - Male sex | 1.94 | 1.83, 2.05 |
|  | - Interaction term | 0.98 | 0.89, 1.08 |

^a^ Adjusted for child parity and year of birth, mother and father’s age, education and income in the year of the child’s birth, the psychiatric history of mother and father prior to the child’s birth and mother and father’s country of origin.

# Table S6: Logistic GEE analyses of the association between maternal smoking cessation in pregnancy and offspring ID

| Model | Coefficient^a^ | O.R. | 95% CI |
| --- | --- | --- | --- |
| Unadjusted | - Stopped smoking in 1st trimester | 1.25 | 1.04, 1.50 |
|  | - Continued smoking after 1st trimester | 2.01 | 1.91, 2.12 |
| Adjusted for confounders ^b^ | - Stopped smoking in 1st trimester | 1.09 | 0.91, 1.32 |
|  | - Continued smoking after 1st trimester | 1.39 | 1.31, 1.47 |

^a^ Reference group is non-smoking mothers
^b^ Adjusted for child sex, parity and year of birth, mother and father’s age, education and income in the year of the child’s birth, the psychiatric history of mother and father prior to the child’s birth and mother and father’s country of origin.

# Table S7: Logistic GEE analyses of the association between dosage of maternal smoking during pregnancy and offspring ID

| Model | Coefficient | O.R. ^a^ | 95% CI |
| --- | --- | --- | --- |
| Unadjusted | - Population averaged | 1.05 | 1.05, 1.06 |
| Adjusted for confounders ^b^ | - Population averaged | 1.03 | 1.02, 1.04 |
| Adjusted for family dosage variable | - Within-family | 1.00 | 0.98, 1.03 |
|  | - Between-family | 1.06 | 1.03, 1.09 |
| Adjusted for confounders ^b^ and family dosage variable | - Within-family | 1.01 | 0.98, 1.03 |
|  | - Between-family | 1.03 | 1.00, 1.06 |

^a^ OR for ID per 1 additional cigarette smoked in pregnancy ^b^ Adjusted for child sex, parity and year of birth, mother and father’s age, education and income in the year of the child’s birth, the psychiatric history of mother and father prior to the child’s birth and mother and father’s country of origin.

# Table S8: Logistic GEE analyses of the association between maternal smoking during pregnancy and offspring ID using a stricter outcome definition

| Model | Coefficient | O.R. | 95% CI |
| --- | --- | --- | --- |
| Unadjusted | - Population averaged | 1.89 | 1.77, 2.01 |
| Adjusted for confounders ^a^ | - Population averaged | 1.35 | 1.26, 1.44 |
| Adjusted for family smoking variable | - Within-family | 0.90 | 0.73, 1.10 |
|  | - Between-family | 2.26 | 1.82, 2.80 |
| Adjusted for confounders ^a^ and family smoking variable | - Within-family | 0.91 | 0.74, 1.13 |
|  | - Between-family | 1.54 | 1.23, 1.93 |

^a^ Adjusted for child sex, parity and year of birth, mother and father’s age, education and income in the year of the child’s birth, the psychiatric history of mother and father prior to the child’s birth and mother and father’s country of origin.

# Table S9: Cox proportional hazards analyses of the association between maternal smoking during pregnancy and time to offspring first diagnosis of ID

| Model | Coefficient | H.R. | 95% CI |
| --- | --- | --- | --- |
| Unadjusted | - Population averaged | 1.87 | 1.78, 1.96 |
| Adjusted for confounders ^a^ | - Population averaged | 1.23 | 1.17, 1.30 |
| Adjusted for family smoking variable | - Within-family | 0.91 | 0.79, 1.05 |
|  | - Between-family | 2.20 | 1.89, 2.57 |
| Adjusted for confounders ^a^ and family smoking variable | - Within-family | 0.89 | 0.76, 1.04 |
|  | - Between-family | 1.44 | 1.21, 1.70 |

^a^ Adjusted for child sex, parity and year of birth, mother and father’s age, education and income in the year of the child’s birth, the psychiatric history of mother and father prior to the child’s birth and mother and father’s country of origin.

# Table S10: Analyses of the association between maternal smoking during pregnancy and offspring ID repeated in restricted cohorts

|  |  | O.R. (95% CI) | | |
| --- | --- | --- | --- | --- |
| Model | Coefficient | Single-child cohort ^a^ | Multiple-child cohort ^b^ | Multiple-child cohort with no missing older siblings ^b^ |
| Unadjusted | - Population averaged | 1.54 (1.38, 1.71) | 1.94 (1.84, 2.04) | 1.87 (1.76, 1.99) |
| Adjusted for confounders ^b^ | - Population averaged | 1.13 (1.01, 1.27) | 1.37 (1.29, 1.45) | 1.33 (1.24, 1.42) |
| Adjusted for family smoking variable | - Within-family | - | 0.91 (0.78, 1.06) | 0.92 (0.77, 1.10) |
|  | - Between-family | - | 2.33 (1.97, 2.75) | 2.23 (1.85, 2.69) |
| Adjusted for confounders ^c^ and family smoking variable | - Within-family | - | 0.96 (0.81, 1.13) | 0.92 (0.76, 1.11) |
|  | - Between-family | - | 1.49 (1.25, 1.79) | 1.52 (1.24, 1.86) |

^a^ Logistic regression model.
^b^ Logistic GEE model with exchangeable covariance structure. ^c^ Adjusted for child sex, mother and father’s age, education and income in the year of the child’s birth, the psychiatric history of mother and father prior to the child’s birth and mother and father’s country of origin.
^d^ Adjusted as for ^c^ but with additional adjustment for child parity.
