# Supplementary material for: Maternal smoking during pregnancy and offspring intellectual disability: sibling analysis in an intergenerational Danish cohort
Source: Psychol Med. 2020 Oct 14;52(10):1847–56. doi: 10.1017/S0033291720003621 (PMC8044256; doi:10.1017/S0033291720003621)
Supplement: Supplementary file 1 [file S0033291720003621sup.zip › S0033291720003621sup002.docx]

Appendices

Maternal smoking during pregnancy and offspring intellectual disability: sibling analysis in an intergenerational Danish cohort

Contents

[A - Methods 2](#_Toc48735662)

[A.1 - Reliability of our smoking measure across registries 2](#_Toc48735663)

[A.2 - Assessment of missing data 2](#_Toc48735664)

[A.3 - Further statistical analysis 2](#_Toc48735665)

[A.3.1 - Secondary analysis 2](#_Toc48735666)

[A.3.2 - Sensitivity analysis 3](#_Toc48735667)

[B - Results 4](#_Toc48735668)

[B.1 - Assessment of missing data 4](#_Toc48735669)

[B.2 - Smoking patterns in the cohort 8](#_Toc48735670)

[B.3 - Patterns of intellectual disability over time 10](#_Toc48735671)

[B.4 - Analyses 11](#_Toc48735672)

[B.4.1 - Secondary analyses 11](#_Toc48735673)

[B.4.2 - Sensitivity analyses 13](#_Toc48735674)

[C - Discussion 14](#_Toc48735675)

[D - References 15](#_Toc48735676)

# Methods

## Reliability of our smoking measure across registries

Where data were available in the NPR, over 99% of smokers identified in the NPR were also recorded as smokers in the MBR, while 0.01% of non-smokers in the NPR were classified as smokers in the MBR (see Table S1; Appendix 2). Smokers, according to the MBR, were more likely to have missing data in the NPR than were non-smokers (OR=1.82; 95% CI=1.80-1.84).

Table A.1-1: Concordance of exposure in Medical Birth Register against National Patient Register.

|  | NPR value | | |
| --- | --- | --- | --- |
| MBR value | Smokers, N(%) | Non-smokers, N(%) | Missing value, N(%) |
| Smoker | 142737 (99.99) | 969 (0.13) | 54671 (26.68) |
| Non-smoker | 14 (0.01) | 718327 (99.87) | 150271 (73.32) |

## Assessment of missing data

Our total sample was reduced by 3.9% (52,157 individuals) due to missing exposure, confounder or covariate variables (n remaining=1,066,989 individuals from 658,335 families). We assessed whether any measured variable predicted whether a value would be excluded from the cohort using complete case logistic regression of a binary indicator for exclusion on each variable separately with no adjustment.

## Further statistical analysis

### Secondary analysis

To assess whether there was a pattern of increasing effect sizes across the severity of diagnosed ID, we performed an unadjusted multinomial logistic GEE model of a categorical variable of highest severity of ID recorded (6 levels: F70, F71, F72, F73, F78/F79, no ID) on maternal smoking during pregnancy with no ID as the reference category. Adjusted analyses could not be performed due to model convergence issues.

We investigated whether there was a pattern of increasing effect sizes across the severity of diagnosed ID by performing unadjusted and confounder adjusted analyses of multinomial logistic GEE model of a categorical variable of comorbidity of ID and ASD (4 levels: ID only, ASD only, ID and ASD, No ID or ASD) on maternal smoking during pregnancy, using no ID or ASD as the reference category. This was repeated for comorbidity of ID with ADHD.

We assessed whether the associations between smoking in pregnancy and offspring ID were influenced by offspring sex we tested an interaction term between maternal smoking and offspring sex. We performed these analyses unadjusted and adjusted for covariates and confounders.

To assess the impact of timing of maternal smoking exposure, we performed unadjusted and confounder adjusted logistic GEE models of ID on the categorical smoking variable of whether the mother gave up smoking during the first trimester of pregnancy. To assess the impact of number of cigarettes (dose), we repeated the four primary analyses models among those who continued to smoke throughout pregnancy and had dosage data available using the number of cigarettes smoked per day as the independent variable. A family-level dosage variable was created by taking the mean number of cigarettes smoked across each pregnancy a mother had for which dosage data were available.

### Sensitivity analysis

We re-ran the primary analyses using a more stringent criterium for ID, defined as having at least two F70-F79 diagnoses recorded in either the DPR or the NPR. A total of 4,452 cases (prevalence = 0.42%) were identified using this definition. We did this in order to examine whether our results were robust to measurement error in the outcome.

We assessed whether differing lengths of follow up between cohort years influenced our conclusions. We therefore repeated the four primary analysis models among sub-cohorts that were grouped by birthyear. We also repeated the four primary analysis models using Cox regression in the survival R package [5, 6]. The Cox models assessed the influence of maternal smoking in pregnancy (binary) on the time in days from birth to the first diagnosis of ID (defined as the first recorded start date of contact with services with a diagnostic code for ID). Individuals who died or emigrated were censored at the date of death or first emigration. Robust standard errors were used for family clusters.

To check for biases arising from smoking patterns in the cohort (see below) we performed additional sensitivity analyses in which we repeated the primary analyses using three restricted cohorts. These cohorts were 1) a cohort of single-child families only (n=175,043; n with ID=1,533), 2) a cohort of multiple-child families only (n=891,946; n with ID=6,517) and 3) a cohort of multiple-child families in which all children were born after the start of the cohort (n=801,109; n with ID=5,007).

# Results

## Assessment of missing data

Results of the missing data assessment can be viewed below in Table B.1-1. Individuals with a diagnosis of intellectual disability had increased odds (OR=1.46; 95% CI=1.34-1.59) of being excluded for having missing data. Individuals whose mother smoked during pregnancy had lower odds (OR=0.67; 95% CI=0.64-0.70) of exclusion for missing data in other covariates compared to those whose mothers did not smoke. Missingness was driven largely by missing maternal smoking data; 74% of those excluded for having missing data (3.5% of the total sample) had missing exposure information (see Table B.1-2). In Table B.1-3 and B.1-4 we present the distribution of missing data in the smoking variable across cohort year group and parity. These tables show that most of the missing data in the smoking variable was in the years 1995-2000 and that there was slightly more missing data for children with greater parity.

Table B.1-1: Descriptive statistics for those included in the main cohort versus those excluded for having missing covariates

| Characteristic | Excluded for missing covariates, N(%) | Included in main cohort, N(%) | O.R.(95% CI) ^a^ | p-value |
| --- | --- | --- | --- | --- |
| Intellectual disability |  |  |  |  |
| -Yes | 571 (1.09) | 8,051 (0.75) | 1.46 (1.34-1.59) | <.001 |
| -No | 51,586 (98.91) | 1,058,938 (99.25) | Ref | - |
| Maternal smoking |  |  |  |  |
| -Yes | 1,798 (13.26) | 198,377 (18.59) | 0.67 (0.64-0.70) | <.001 |
| -No | 11,759 (86.74) | 868,612 (81.41) | Ref | - |
| Maternal age |  |  |  |  |
| -<20 | 1,044 (2.01) | 14,682 (1.38) | 1.49 (1.40-1.59) | <.001 |
| -20-24 | 7,416 (14.25) | 125,515 (11.76) | 1.24 (1.21-1.28) | <.001 |
| -25-29 | 17,455 (33.53) | 366,516 (34.35) | Ref | - |
| -30-34 | 17,229 (33.10) | 380,506 (35.66) | 0.95 (0.93-0.97) | <.001 |
| -35+ | 8,913 (17.12) | 179,770 (16.85) | 1.04 (1.01-1.07) | .003 |
| Paternal age |  |  |  |  |
| -<20 | 254 (0.49) | 4,238 (0.40) | 1.24 (1.09-1.41) | .001 |
| -20-24 | 3,383 (6.51) | 61,559 (5.77) | 1.13 (1.09-1.18) | <.001 |
| -25-29 | 12,811 (24.64) | 264,433 (24.78) | Ref | - |
| -30-34 | 18,145 (34.90) | 392,876 (36.82) | 0.95 (0.93-0.98) | <.001 |
| -35+ | 17,397 (33.46) | 343,882 (32.23) | 1.04 (1.02-1.07) | <.001 |
| Maximum parental education |  |  |  |  |
| -Primary | 5,594 (13.17) | 107,273 (10.05) | Ref | - |
| -General/Vocational | 19,380 (45.64) | 462,159 (43.31) | 0.80 (0.78-0.83) | <.001 |
| -Higher | 17,486 (41.18) | 497,557 (46.63) | 0.67 (0.65-0.70) | <.001 |
| Parental income decile |  |  |  |  |
| -1 | 9,483 (18.19) | 102,424 (9.60) | 2.20 (2.12-2.28) | <.001 |
| -2 | 6,164 (11.82) | 105,749 (9.91) | 1.38 (1.33-1.44) | <.001 |
| -3 | 5,361 (10.28) | 106,556 (9.99) | 1.19 (1.15-1.24) | <.001 |
| -4 | 5,020 (9.63) | 106,894 (10.02) | 1.11 (1.07-1.16) | <.001 |
| -5 | 4,526 (8.68) | 107,385 (10.06) | Ref | - |
| -6 | 4,595 (8.81) | 107,326 (10.06) | 1.02 (0.97-1.06) | .463 |
| -7 | 4,523 (8.67) | 107,392 (10.06) | 1.00 (0.96-1.04) | .973 |
| -8 | 4,307 (8.26) | 107,607 (10.09) | 0.95 (0.91-0.99) | .017 |
| -9 | 3,970 (7.61) | 107,946 (10.12) | 0.87 (0.84-0.91) | <.001 |
| -10 | 4,196 (8.05) | 107,710 (10.09) | 0.92 (0.89-0.96) | <.001 |
| Maternal country of origin |  |  |  |  |
| -Denmark | 35,852 (71.82) | 928,190 (86.99) | Ref | - |
| -Africa | 1,839 (3.68) | 17,130 (1.61) | 2.78 (2.65-2.92) | <.001 |
| -Americas | 533 (1.07) | 6,013 (0.56) | 2.29 (2.10-2.51) | <.001 |
| -Europe | 5,341 (10.70) | 46,295 (4.34) | 2.99 (2.90-3.08) | <.001 |
| -Middle East | 2,335 (4.68) | 25,403 (2.38) | 2.38 (2.28-2.49) | <.001 |
| -Oceana | 2,284 (4.58) | 29,118 (2.73) | 2.03 (1.94-2.12) | <.001 |
| -Scandinavia | 1,738 (3.48) | 14,840 (1.39) | 3.03 (2.88-3.19) | <.001 |
|  |  |  |  |  |
| Paternal country of origin |  |  |  |  |
| -Denmark | 35,622 (72.28) | 931,110 (87.27) | Ref | - |
| -Africa | 1,829 (3.71) | 18,980 (1.78) | 2.52 (2.40-2.65) | <.001 |
| -Americas | 443 (0.90) | 5,626 (0.53) | 2.06 (1.87-2.27) | <.001 |
| -Europe | 5,393 (10.94) | 50,035 (4.69) | 2.82 (2.73-2.90) | <.001 |
| -Middle East | 2,521 (5.12) | 30,348 (2.84) | 2.17 (2.08-2.26) | <.001 |
| -Oceana | 1,903 (3.86) | 20,046 (1.88) | 2.48 (2.36-2.60) | <.001 |
| -Scandinavia | 1,572 (3.19) | 10,844 (1.02) | 3.79 (3.59-4.00) | <.001 |
| Maternal psychiatric history |  |  |  |  |
| -Affective disorder |  |  |  |  |
| -Yes | 755 (1.45) | 18,343 (1.72) | 0.84 (0.78-0.90) | <.001 |
| -No | 51,402 (98.55) | 1,048,646 (98.28) | Ref | - |
| -Anxiety disorder |  |  |  |  |
| -Yes | 1,659 (3.18) | 39,859 (3.74) | 0.85 (0.81-0.89) | <.001 |
| -No | 50,498 (96.82) | 1,027,130 (96.26) | Ref | - |
| -Psychotic disorder |  |  |  |  |
| -Yes | 263 (0.50) | 5,072 (0.48) | 1.06 (0.94-1.20) | .350 |
| -No | 51,894 (99.50) | 1,061,917 (99.52) | Ref | - |
| -Substance use disorder |  |  |  |  |
| -Yes | 738 (1.41) | 18,020 (1.69) | 0.84 (0.78-0.90) | <.001 |
| -No | 51,419 (98.59) | 1,048,969 (98.31) | Ref | - |
| Paternal psychiatric history |  |  |  |  |
| -Affective disorder |  |  |  |  |
| -Yes | 319 (0.61) | 6,708 (0.63) | 0.97 (0.87-1.09) | .630 |
| -No | 51,838 (99.39) | 1,060,281 (99.37) | Ref | - |
| -Anxiety disorder |  |  |  |  |
| -Yes | 842 (1.61) | 1,7403 (1.63) | 0.99 (0.92-1.06) | .769 |
| -No | 51,315 (98.39) | 1,049,586 (98.37) | Ref | - |
| -Psychotic disorder |  |  |  |  |
| -Yes | 283 (0.54) | 4,977 (0.47) | 1.16 (1.03-1.31) | .013 |
| -No | 51,874 (99.46) | 1,062,012 (99.53) | Ref | - |
| -Substance use disorder |  |  |  |  |
| -Yes | 1,048 (2.01) | 25,539 (2.39) | 0.84 (0.79-0.89) | <.001 |
| -No | 51,109 (97.99) | 1,041,450 (97.61) | Ref | - |
| Child sex |  |  |  |  |
| -Female | 25,306 (48.52) | 519,856 (48.72) | Ref | - |
| -Male | 26,851 (51.48) | 547,133 (51.28) | 1.01 (0.99-1.03) | .365 |
| Parity |  |  |  |  |
| -0 | 22,836 (43.79) | 461,759 (43.28) | Ref | - |
| -1 | 18,321 (35.13) | 400,499 (37.54) | 0.93 (0.91-0.94) | <.001 |
| -2 | 7,423 (14.23) | 152,496 (14.29) | 0.98 (0.96-1.01) | .247 |
| -3+ | 3,569 (6.84) | 52,235 (4.9) | 1.38 (1.33-1.43) | <.001 |
|  |  |  |  |  |
| Cohort year |  |  |  |  |
| -1995-1997 | 13,575 (26.03) | 180,394 (16.91) | Ref | - |
| -1998-2000 | 11,862 (22.74) | 182,132 (17.07) | 0.87 (0.84-0.89) | <.001 |
| -2001-2003 | 6,704 (12.85) | 177,831 (16.67) | 0.50 (0.49-0.52) | <.001 |
| -2004-2006 | 6,070 (11.64) | 180,674 (16.93) | 0.45 (0.43-0.46) | <.001 |
| -2007-2009 | 7,310 (14.02) | 178,532 (16.73) | 0.54 (0.53-0.56) | <.001 |
| -2010-2012 | 6,636 (12.72) | 167,426 (15.69) | 0.53 (0.51-0.54) | <.001 |

^a^ Odds Ratio for exclusion from the cohort due to missing covariates.

Table B.1-2: Number of observations with missing data in each variable

| Variable | Number of missing values | % of those excluded ^a,b^ | % of total sample ^a,c^ |
| --- | --- | --- | --- |
| Maternal smoking in pregnancy | 38,600 | 74.01 | 3.45 |
| Highest parental education | 9,697 | 18.59 | 0.87 |
| Paternal country of origin | 2,874 | 5.51 | 0.26 |
| Maternal country of origin | 2,235 | 4.29 | 0.20 |
| Paternal age | 167 | 0.32 | 0.01 |
| Maternal age | 100 | 0.19 | 0.01 |
| Income decile | 12 | 0.02 | <0.01 |
| Parity | 8 | 0.02 | <0.01 |

^a^ Groups not mutually exclusive so percentages may not add to 100%.
^b^ Percentage denominator equals 93,190
^c^ Percentage denominator equals 1,119,146

Table B.1-3: Distribution of missing smoking data by each cohort year group

| Year group | Excluded for missing smoking data, N(%) ^a^ | Included in main cohort, N(%) ^b^ |
| --- | --- | --- |
| 1995-1997 | 10,770 (27.90) | 183,199 (16.95) |
| 1998-2000 | 9,652 (25.01) | 184,342 (17.06) |
| 2001-2003 | 4,865 (12.60) | 179,670 (16.63) |
| 2004-2006 | 4,478 (11.60) | 182,266 (16.87) |
| 2007-2009 | 5,041 (13.06) | 180,801 (16.73) |
| 2010-2012 | 3,794 (9.83) | 170,268 (15.76) |

^a^ Percentage denominator equals 38,600
^b^ Percentage denominator equals 1,066,989

Table B.1-4: Distribution of missing smoking data by parity

| Parity | Excluded for missing smoking data, N(%) ^a^ | Included in main cohort, N(%) ^b^ |
| --- | --- | --- |
| 0 | 16,771 (43.45) | 467,824 (43.30) |
| 1 | 14,066 (36.44) | 404,754 (37.46) |
| 2 | 5,496 (14.24) | 154,423 (14.29) |
| 3+ | 2,265 (5.87) | 53,539 (4.95) |

^a^ Percentage denominator equals 38,600
^b^ Percentage denominator equals 1,066,989

## Smoking patterns in the cohort

As parity increased, changes in smoking from one pregnancy to the next were less likely to occur (see Table B.1-1). For children with parity>1, an index child was less likely to have ID if their mother had smoked in neither the index pregnancy or the previous pregnancy and was more likely to have ID if their mother had smoked in either pregnancy (see Table B.1-2).

We present a cross tabulation of the family-level exposure variable against case status for ID in Table B.1-3. Exposure discordance within a family (i.e. a family-level exposure value not equal to 0 or 1) was present for 66,798 individuals (6.3% of the primary analysis sample), across 28,748 different families (4.4% of all families in the primary analysis sample).

207,121 families (31.46% of all families in the primary analysis sample) had a child born before the cohort start date. Information on such children was not observed and so not included in the family-level variable; the family-level variable in such families may contain error as a result. Families with a child excluded for being born before the cohort start date were less likely to have exposure discordance and were more likely to have all siblings exposed than families in which all children were born after the cohort start date (see Table B.1-4).

Only-children were more likely to be exposed to smoking in pregnancy than the first-born child of families with multiple children in the cohort (see Table B.1-5). This pattern held true when stratifying separately on birthyear and maternal age indicating that single and multiple-child families may not be comparable.

Table B.1-1: Distribution of changes in smoking from one pregnancy to the next

|  | Parity, N(%) | | | | | |
| --- | --- | --- | --- | --- | --- | --- |
|  | 0/1 | 1/2 | 2/3 | 3/4 | 4/5 | 5/6 |
| Smoked in neither | 215,006 (81.84) | 71,405 (81.69) | 16,330 (76.71) | 4,309 (75.84) | 1,539 (77.96) | 662 (80.63) |
| Smoked in both | 26,027 (9.91) | 10,016 (11.46) | 3,401 (15.98) | 1,014 (17.85) | 330 (16.72) | 124 (15.10) |
| Started smoking | 6,710 (2.55) | 2,274 (2.60) | 595 (2.80) | 147 (2.59) | 46 (2.33) | 15 (1.83) |
| Stopped smoking | 14,982 (5.70) | 3,712 (4.25) | 962 (4.52) | 212 (3.73) | 59 (2.99) | 20 (2.44) |

Table B.1-2: Distribution of changes in smoking from one pregnancy to the next separated by the exposure and outcome status of the latter pregnancy

|  | Maternal smoking in pregnancy, N(%) | | Intellectual Disability, N(%) | |
| --- | --- | --- | --- | --- |
|  | No | Yes | No | Yes |
| Smoked in neither | 315,170 (93.93) | - | 313,663 (81.49) | 1507 (68.01) |
| Smoked in both | - | 41,611 (80.66) | 41,118 (10.68) | 493 (22.25) |
| Started smoking | - | 9,975 (19.34) | 9,892 (2.57) | 83 (3.75) |
| Stopped smoking | 20,359 (6.07) | - | 20,226 (5.25) | 133 (6.00) |

Table B.1-3: Proportions of ID in each level of the family level exposure variable

|  | Siblings in a family exposed to smoking in pregnancy, N(%) | | | | | |
| --- | --- | --- | --- | --- | --- | --- |
|  | None | | Some | | All | Total |
| Counts for individuals |  |  | |  | |  |
| -No ID | 829,246 (99.39) | 66,288 (99.24) | | 163,404 (98.54) | | 1,058,938 (99.25) |
| -ID | 5,118 (0.61) | 510 (0.76) | | 2,423 (1.46) | | 8,051 (0.75) |
| - Total | 834,364 (100.0) | 66,798 (100.0) | | 165,827 (100.0) | | 1,066,990 (100.0) |
| Counts for families |  |  | |  | |  |
| -No ID | 500,808 (99.01) | 28,255 (98.29) | | 121,407 (98.08) | | 650,470 (98.81) |
| -At least 1 case of ID | 4,999 (0.99) | 493 (1.71) | | 2,373 (1.92) | | 7,865 (1.19) |
| -Total | 505,807 (100.0) | 28,748 (100.0) | | 123,780 (100.0) | | 658,335 (100.0) |

Table B.1-4: Distribution of family smoking variable among families with children born before the cohort start date versus families with children all born after the cohort start date

| Family level exposure value | Families with excluded older siblings, N(%) | Families with first born included in cohort, N(%) |
| --- | --- | --- |
| 0 (smoked in no pregnancies) | 145,837 (70.41) | 359,970 (79.78) |
| 0.01-0.33 | 84 (0.04) | 459 (0.10) |
| 0.34-0.66 | 3,924 (1.89) | 21,278 (4.72) |
| 0.67-0.99 | 449 (0.22) | 2,554 (0.57) |
| 1 (smoked in all pregnancies) | 56,827 (27.44) | 66,953 (14.84) |

Table B.1-5: Distribution of smoking among only children and first-born children

|  | N(%) | |
| --- | --- | --- |
|  | Only child | First born child |
| Non-smoker | 134,458 (76.81) | 242,190 (84.47) |
| Smoker | 40,585 (23.19) | 44,526 (15.53) |

## Patterns of intellectual disability over time

By taking a snapshot of prevalence by age in 2017, Figure B.3-1 shows that the prevalence increased with age up to an age of 18 where it started to level off. Prevalence was higher among males than females at all ages. The prevalence of ID at ages 6-18 years of age for each cohort year is displayed in Figure B.3-2. Prevalence remained approximately steady at each age over time, though some cohort years (e.g. 1999) have slightly greater prevalence at all ages.

Figure B.3-1: Plot of the prevalence of intellectual disability for each age in 2017.


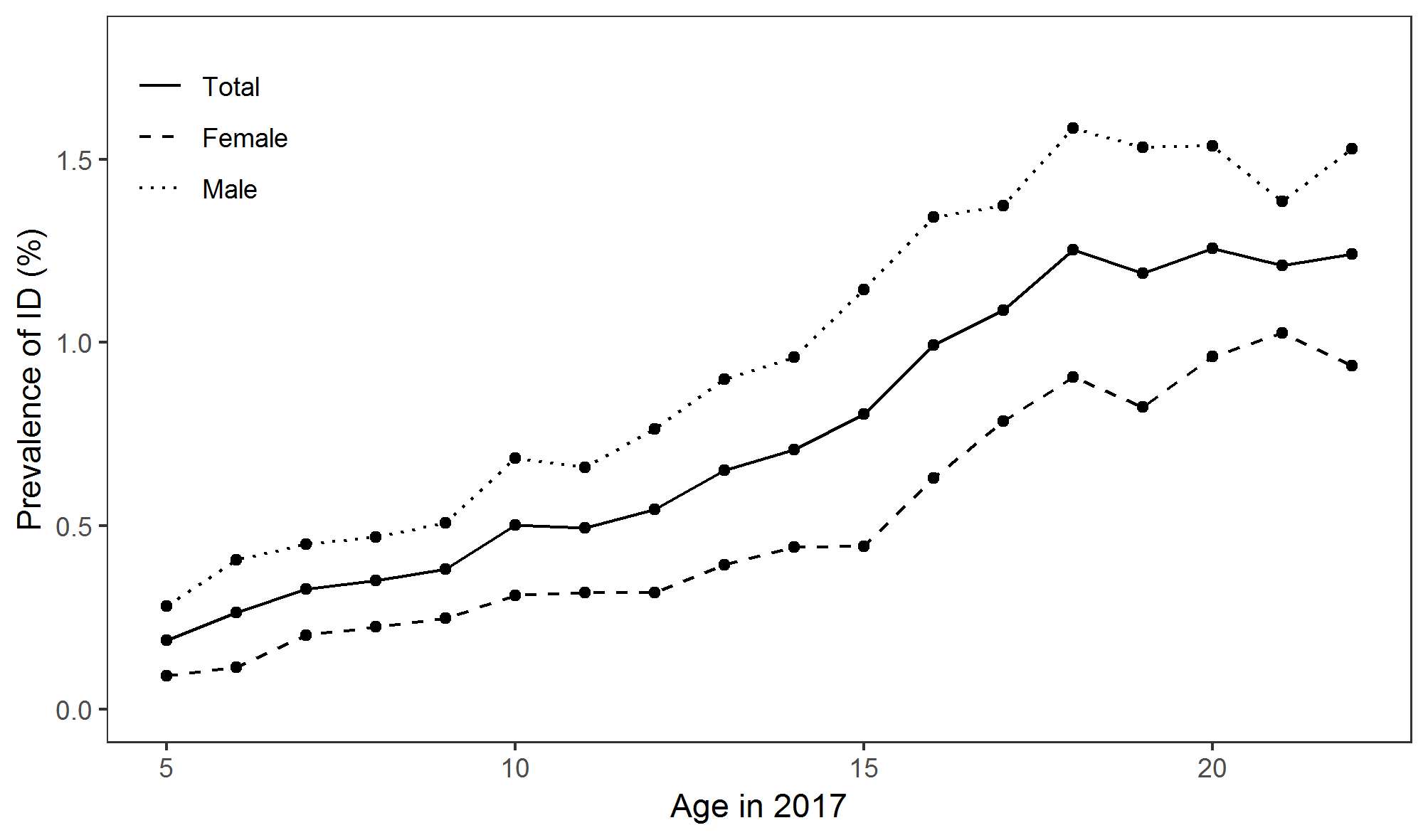


Figure B.3-2: Plot of the prevalence of intellectual disability at ages 6-18 for each cohort year.


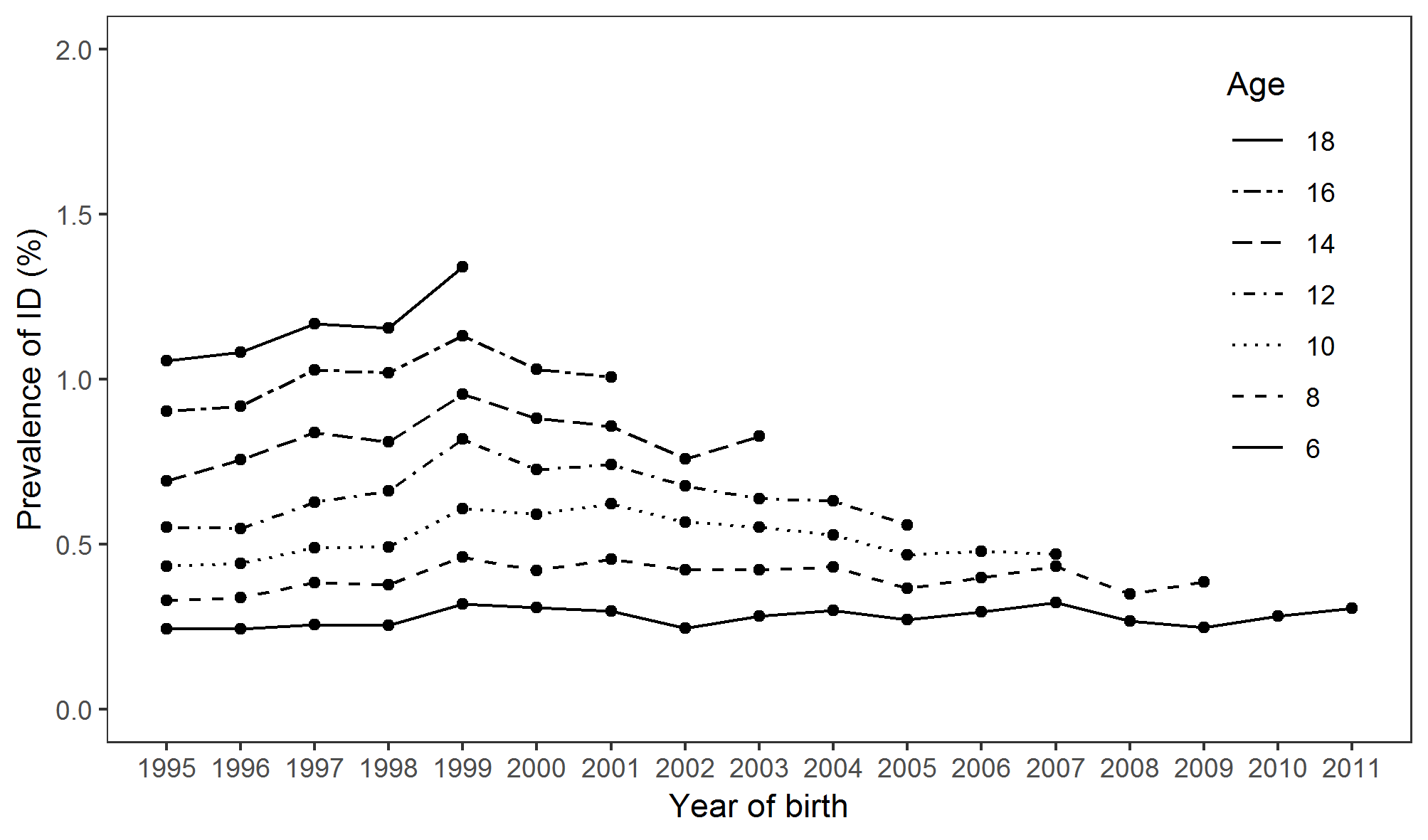


## Analyses

### Secondary analyses

The distribution of ID across ICD-10 severity codes, comorbidities with ASD and ADHD, sex, smoking cessation behaviours, and dosage groups are presented in Table B.4.1-1 below. The highest severity diagnosis received by cohort members was most often mild or unspecified ID (F70 and F78/9). There was a higher prevalence of ID among those with ASD or ADHD than those without. The prevalence of ID was higher among those whose mother continued to smoke after the 1^st^ trimester than those whose mothers quit during the first trimester or who did not smoke. A pattern of increasing prevalence of ID with greater numbers of cigarettes smoked per day during pregnancy was also observed.

In unadjusted analyses mild, moderate and unspecified ID were all associated with maternal smoking during pregnancy (Table B.4.1-2). The odds for the association with mild ID were greater than those for moderate ID.

Analyses for comorbidity with ASD and ADHD are presented in Table B.4.1-3 and Table B.4.1-4 respectively. In unadjusted analyses, all combinations of ID with ASD and combinations of ID with ADHD showed an association of increased odds among those exposed to maternal smoking in pregnancy. Following adjustment for confounders and family averaged exposure, the within-family effects for all comorbidity combinations were null.

For the sex difference analysis the interaction term was null in unadjusted and adjusted analyses (see supplementary Table S5). This suggests that there is no difference in effect size between male and female offspring.

In our timing analysis both the unadjusted and adjusted models showed that, relative to non-smokers, the OR for ID was greater among those who continued smoking during pregnancy than those who quit during the first trimester (see supplementary Table S6).

The dosage analysis showed a 5% increase in odds of ID for each additional cigarette smoked per day (see supplementary Table S7). This effect was attenuated to a 3% increase following adjustment for confounders. A null within-family effect was found in all models that adjusted for the family averaged dose. The between-family effect was a 6% increase in odds per cigarette smoked per day on average across pregnancies, holding fixed the number smoked in each individual pregnancy. This attenuated to a 3% increase after adjustment for confounders.

Table B.4.1-1: Distribution of ID across secondary analysis categories

| Category | ID, N(%) | No ID, N(%) |
| --- | --- | --- |
| Severity analysis ^a^ |  |  |
| - F70 | 4,436 (55.10) | - |
| - F71 | 1,288 (16.00) | - |
| - F72 | 451 (5.60) | - |
| - F73 | 176 (2.19) | - |
| - F78/9 | 1,700 (21.11) | - |
|  |  |  |
| Comorbidity analysis ^b^ |  |  |
| - ASD |  |  |
| - Yes | 2,840 (13.58) | 18,075 (86.42) |
| - No | 5,211 (0.50) | 1,040,863 (99.50) |
| - ADHD |  |  |
| - Yes | 2,033 (9.87) | 18,565 (90.13) |
| - No | 6,018 (0.58) | 1,040,373 (99.42) |
|  |  |  |
| Sex difference analysis ^b^ |  |  |
| - Females | 2,680 (0.52) | 517,176 (99.48) |
| - Males | 5,371 (0.98) | 541,762 (99.02) |
| Timing analysis ^b^ |  |  |
| - Non-smoker | 4,469 (0.57) | 775,546 (99.43) |
| - Stopped smoking during the first trimester | 115 (0.67) | 16,972 (99.33) |
| - Continued smoking after the first trimester | 1,986 (1.33) | 147,083 (98.67) |
| Dosage analysis ^b^ |  |  |
| - Smoked up to 5 cigarettes per day | 399 (0.92) | 43,169 (99.08) |
| - Smoked 6-10 cigarettes per day | 713 (1.35) | 52,263 (98.65) |
| - Smoked 11-20 cigarettes per day | 642 (1.63) | 38,731 (98.37) |
| - Smoked more than 20 cigarettes per day | 144 (2.73) | 5,134 (97.27) |

^a^ Column percentage
^b^ Row percentage

Table B.4.1-2: Multinomial logistic GEE analyses of the association between maternal smoking in pregnancy and each ID severity category

|  | Unadjusted analyses | |
| --- | --- | --- |
| Coefficient | O.R. | 95% CI |
| - F70 – Mild | 2.17 | 2.04 - 2.31 |
| - F71 – Moderate | 1.53 | 1.35 - 1.73 |
| - F72 – Severe | 1.20 | 0.96 - 1.50 |
| - F73 – Profound | 1.37 | 0.97 - 1.94 |
| - F78/F79 – Other/unspecified | 1.77 | 1.59 - 1.97 |

All odds ratios are relative to the group with no ID

### Sensitivity analyses

Our replication of the primary analyses with a stricter ID outcome definition are presented in supplementary Table S8. The results closely resemble those of the primary analyses though ORs were slightly smaller, and CIs were slightly wider.

We assessed whether length of follow up influenced the results of our analyses. The four primary analyses models, repeated over cohort year groups, show that in all but year groups 1995-1997 and 2010-2012 there was a null within-family effect and a between-family effect displaying increased odds of ID with an increased proportion of pregnancies in which a mother smoked (see Figure 2 in the main article; the underlying data for this figure can be found in Table B.4.2-1 below). In the 1995-1997 group there was a null between-family effect while in the 2010-2012 group the standard errors of both effects were large, suggesting low power to detect an effect. The cox proportional hazards models (see supplementary Table S9) provide results that are consistent with those obtained from the logistic GEE models.

In our restricted cohort analyses (see supplementary Table S10) the confounder adjusted OR of the single-child cohort was smaller than that of the total cohort. The results in the multiple-child cohort and the multiple-child cohort with no missing older siblings did not differ meaningfully from those of the total cohort.

Table B.4.2-1: Logistic GEE analyses of the association between maternal smoking during pregnancy and offspring ID repeated in each year group category

|  |  | | O.R. (95% CI) | | | | | | |
| --- | --- | --- | --- | --- | --- | --- | --- | --- | --- |
| Model | | Coefficient | | 1995-1997 | 1998-2000 | 2001-2003 | 2004-2006 | 2007-2009 | 2010-2012 |
| Unadjusted | | - Population averaged | | 1.82  (1.67, 1.98) | 1.73  (1.58, 1.89) | 2.12  (1.90, 2.36) | 2.17  (1.90, 2.49) | 2.04  (1.72, 2.41) | 1.93  (1.54, 2.44) |
| Adjusted for confounders^a^ | | - Population averaged | | 1.28  (1.17, 1.41) | 1.21  (1.10, 1.33) | 1.50  (1.33, 1.69) | 1.49  (1.28, 1.72) | 1.44  (1.19, 1.73) | 1.50  (1.17, 1.94) |
| Adjusted for family smoking variable | | - Within-family | | 0.98  (0.69, 1.40) | 0.74  (0.56, 0.97) | 0.95  (0.71, 1.28) | 0.99  (0.68, 1.43) | 0.89  (0.56, 1.42) | 1.72  (0.70, 4.22) |
|  |  | - Between-family | | 1.93  (1.35, 2.78) | 2.55  (1.91, 3.40) | 2.46  (1.78, 3.39) | 2.44  (1.63, 3.64) | 2.51  (1.53, 4.11) | 1.13  (0.45, 2.87) |
| Adjusted for confounders ^a^ and family smoking variable | | - Within-family | | 1.03  (0.71, 1.48) | 0.78  (0.58, 1.05) | 1.01  (0.73, 1.39) | 0.97  (0.65, 1.44) | 0.80  (0.49, 1.31) | 1.46  (0.57, 3.73) |
|  |  | - Between-family | | 1.27  (0.87, 1.85) | 1.62  (1.19, 2.21) | 1.57  (1.11, 2.23) | 1.63  (1.06, 2.49) | 1.93  (1.14, 3.29) | 1.03  (0.39, 2.74) |

^a^ Adjusted for child sex and parity, mother and father’s age, education and income in the year of the child’s birth, the psychiatric history of mother and father prior to the child’s birth and mother and father’s country of origin.

# Discussion

In secondary analyses we found unadjusted associations with milder and unspecified forms of ID but not more severe forms which may suggest that milder ID is more susceptible to environmental exposures than more severe ID. We also found an effect of exposure timing that could suggest a sensitive period of exposure. Both results need to be treated with caution. Severity may have been measured with substantial error as the unspecified severity group made up the second largest of all groups. Every case in this unspecified diagnosis group belongs to one of the specified diagnosis categories, however, the proportion in each is unclear. Each association estimate in the severity analysis may therefore be biased to an unknown extent. Regarding the timing analysis, we are not aware of a parameterization that would allow the separation of effects into within and between-family effects for a categorical variable and so were not able to control for family-level differences in smoking cessation during pregnancy. The effect of exposure timing may therefore simply reflect familial confounding. Further, smoking cessation during pregnancy is commonly misreported [7] and may be attempted multiple times. Our measure of smoking cessation may therefore not adequately capture the timing of exposure during pregnancy.

# References

1. Pedersen, C.B., et al., *A comprehensive nationwide study of the incidence rate and lifetime risk for treated mental disorders.* JAMA Psychiatry, 2014. **71**(5): p. 573-81.

2. Bliddal, M., et al., *The Danish Medical Birth Register.* Eur J Epidemiol, 2018. **33**(1): p. 27-36.

3. Pedersen, C.B., et al., *The iPSYCH2012 case-cohort sample: new directions for unravelling genetic and environmental architectures of severe mental disorders.* Mol Psychiatry, 2018. **23**(1): p. 6-14.

4. Begg, M.D. and M.K. Parides, *Separation of individual-level and cluster-level covariate effects in regression analysis of correlated data.* Stat Med, 2003. **22**(16): p. 2591-602.

5. Therneau, T., *A package for Survival Analysis in S*. 2015.

6. Therneau, T.M. and P.M. Grambsch, *Modeling survival data : extending the Cox model*. Statistics for biology and health. 2000, New York: Springer. xiii, 350 p.

7. George, L., et al., *Self-reported nicotine exposure and plasma levels of cotinine in early and late pregnancy.* Acta Obstetricia Et Gynecologica Scandinavica, 2006. **85**(11): p. 1331-1337.
